# The effect of female breast surface area on heat‐activated sweat gland density and output

**DOI:** 10.1113/EP091850

**Published:** 2024-06-07

**Authors:** Hannah Blount, Alessandro Valenza, Jade Ward, Silvia Caggiari, Peter R. Worsley, Davide Filingeri

**Affiliations:** ^1^ ThermosenseLab, Skin Sensing Research Group, School of Health Sciences The University of Southampton Southampton UK; ^2^ Sport and Exercise Sciences Research Unit, SPPEFF Department University of Palermo Palermo Italy; ^3^ PressureLab, Skin Sensing Research Group, School of Health Sciences The University of Southampton Southampton UK

**Keywords:** breast, exercise, female, morphology, sweat

## Abstract

Female development includes significant morphological changes across the breast. Yet, whether differences in breast surface area (BrSA) modify sweat gland density and output remains unclear. The present study investigated the relationship between BrSA and sweat gland density and output in 22 young to middle‐aged women (28±10 years) of varying breast sizes (BrSA range: 147–561 cm^2^) during a submaximal run in a warm environment (32 ±0.6°C; 53 ±1.7% relative humidity). Local sweat gland density and local sweat rate (LSR) above and below the nipple and at the bra triangle were measured. Expired gases were monitored for the estimation of evaporative requirements for heat balance (*E*_req_, in W/m^2^). Associations between BrSA and (i) sweat gland density; (ii) LSR; and (iii) sweat output per gland for the breast sites were determined via correlation and regression analyses. Our results indicated that breast sweat gland density decreased linearly as BrSA increased (*r* = −0.76, *P *< 0.001), whereas sweat output per gland remained constant irrespective of BrSA (*r* = 0.29, *P *= 0.28). This resulted in LSR decreasing linearly as BrSA increased (*r* = −0.62, *P *= 0.01). Compared to the bra triangle, the breast had a 64% lower sweat gland density (*P *< 0.001), 83% lower LSR (*P *< 0.001) and 53% lower output per gland (*P *< 0.001). BrSA (*R* ^2^ = 0.33, *P *= 0.015) explained a greater proportion of variance in LSR than *E*_req_ (in W/m^2^) (*R* ^2^ = 0.07, *P *= 0.538). These novel findings extend the known relationship between body morphology and sweat gland density and LSR, to the female breast. This knowledge could innovate user‐centred design of sports bras by accommodating breast size‐specific needs for sweat management, skin wetness perception and comfort.

## Abstract (highlights)

- **What is the central question of this study?**
  How do differences in breast surface area impact sweat gland density and output across the breast of healthy women during running in the heat?
- **What is the main finding and its importance?**
  There was a novel relationship between breast morphology and sweat gland density and output such that gland density and local sweat rates decreased linearly as breast surface area increased. Furthermore, the breast had lower sweat gland density and output than the bra triangle. This knowledge could inform user‐centred design of sports bras through a better understanding of breast size‐specific thermal needs.

## 1 INTRODUCTION

Following behavioural thermoregulation, the production and evaporation of sweat from the skin surface is the human body's principal and most powerful method of heat loss during exercise and heat stress (Havenith, 1999; Havenith et al., 2008). By 2 years of age, our skin contains 2–5 million sweat glands, which are the main end organs supporting sweat production and secretion onto the skin (Kuno, 1956). The number of sweat glands does not appear to change beyond this age, and hence sweat gland density decreases with skin expansion during physical growth and musculoskeletal maturation (Kuno, 1956; Szabo, 1958).

In contrast to development in males, female development includes significant morphological changes across specific body parts, such as the breast. Female breast development, and the resulting breast surface area (BrSA), can vary greatly due to genetic factors, body mass index, and energy intake early in life (Trichopoulos & Lipman, 1992; Wade et al., 2010). However, it is unknown whether regional sweat gland density further decreases as breasts grow.

Understanding the relationship between BrSA and sweat gland density over this body part is important, as sweat gland density and output per gland may impact local sweat rates (LSR) (Kondo et al., 2001), and consequently the distribution of sweat across the breast during exercise. LSR across the breast have been previously measured by Smith and Havenith (2012), who found most regional differences between the bra triangle (i.e., higher LSR) and the breast (i.e., lower LSR). However, the work of Smith and Havenith (2012) did not address the impact of breast size as a variable in modulating LSR. The majority of women use sports bras as an essential item of clothing during exercise to support the breast and reduce the amount of breast movement (Gehlsen & Albohm, 1980; Lorentzen & Lawson, 1987; Scurr et al., 2010) and breast discomfort (Brown et al., 2014). Differences in the distribution of sweat across breasts of different surface areas could modify the pattern of sweat accumulation in sport bras, which could in turn impact breasts’ heat balance and comfort during exercise heat stress in women (Ayres et al., 2013; Gorea et al., 2020).

The number of women taking part in sport has increased considerably over the last 50 years with data indicating almost 50% of women worldwide are now interested in sport; yet, kit and equipment can be a barrier to exercise participation for many women (Sky‐Sports, 2023). Furthermore, women continue to be largely unrepresented in heat stress research, with a recent review highlighting that only 12%–18% of participants in thermoregulation research over the last decade were female (Hutchins et al., 2021). Hence, increasing our fundamental understanding of sweat gland distribution and function at the breast may support innovation in women's sportswear design to remove barriers to an active lifestyle across the lifespan, with related health benefits for the global female population.

The primary aim of this study was to investigate the relationship between BrSA and sweat gland density across the female breast in a cohort of healthy women of varying breast sizes. This was assessed during a submaximal run in a warm environment. Our primary hypothesis was that sweat gland density over the breast would decrease with increasing BrSA. The secondary aim of this study was to investigate the relationship between BrSA and sweat output per gland and associated LSR across the female breast in the same cohort of women. Our secondary hypothesis was that sweat output per gland would not vary with BrSA, thereby leading to decreasing LSR at the breast with increasing BrSA.

## 2 METHODS

### 2.1 Ethical approval

This study was approved by the University of Southampton Ethics Committee (approval no. 79007). All participants provided written informed consent prior to testing. The study conformed to the ethical standards set by the *Declaration of Helsinki*, except for registration in a database.

### 2.2 Participants

The study involved a convenience sampling approach of women varying in BrSA. Due to the non‐linear association between BrSA and bra size (i.e., the latter being the most intuitive way of determining one's breast size for eligibility purposes) we opted for the recruitment of five to six women for each of four bra‐size categories, namely, small, medium, large and extra‐large. This purposeful recruitment was used to achieve a wide range of BrSA. Women are indeed typically familiar with such a classification of bra size, and this would have aided participant recruitment. As a result, we expected to recruit a total sample size of 20–24 healthy young and middle‐aged women, which is in line with most studies in human thermoregulation (Buono & Connolly, 1992; Havenith et al., 2008; Kondo et al., 2001).

Participant recruitment resulted in 22 females taking part in the study (age: 27.7±9.6 years; weight: 72.2±12.7 kg; height: 170.4 ±4.8 cm) (Table 1). Inclusion criteria included physically active women (i.e., performing 30 min regular exercise of moderate intensity at least 3 days each week), free from musculoskeletal or neurological disease, not under any pharmacological treatment, with standard breast tissue type (i.e., no implants, reductions or mastectomy) and who fit size small, medium, large or extra‐large sports bras. They were also instructed to refrain from: (i) performing strenuous exercise in the 48 h preceding testing; (ii) consuming caffeine or alcohol in the 24 h preceding testing; (iii) consuming food in the 3 h prior to testing; and (iv) applying creams or gels to the chest region. Nineteen participants were well spread across a typical 28‐day menstrual cycle (mean day of cycle: 13.6 ± 8.2) and three participants presented irregular periods at the time of the study.

**TABLE 1:** Participant demographics (*n* = 22).

	Age (years)		BMI (kg/m^2^)		BSA (m^2^)		BrSA (c m^2^)	
Bra size	Mean	Min–Max	Mean	Min–Max	Mean	Min–Max	Mean	Min–Max
Small (*n* = 6)	23.3	18–30	21.8	19.5–25.5	1.68	1.60–1.75	168	147–230
Medium (*n* = 5)	22.8	19–27	23.7	21.6–26.5	1.77	1.66–1.87	246	204–288
Large (*n* = 6)	30.2	20–42	24.4	21.5–29.1	1.85	1.70–1.94	316	174–402
X‐Large (*n* = 5)	34.8	21–55	29.9	25.9–35.1	2.05	1.88–2.21	459	300–562

Abbreviations: BMI, body mass index; BrSA, breast surface area; BSA, body surface area.

### 2.3 Experimental design

**FIGURE 1:**
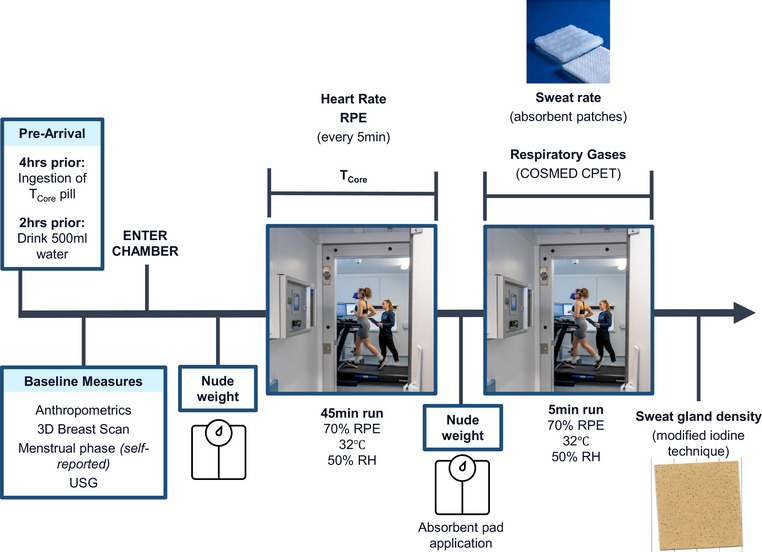
Schematic representation of experimental design. RH, relative humidity; USG, urine specific gravity.

**FIGURE 2:**
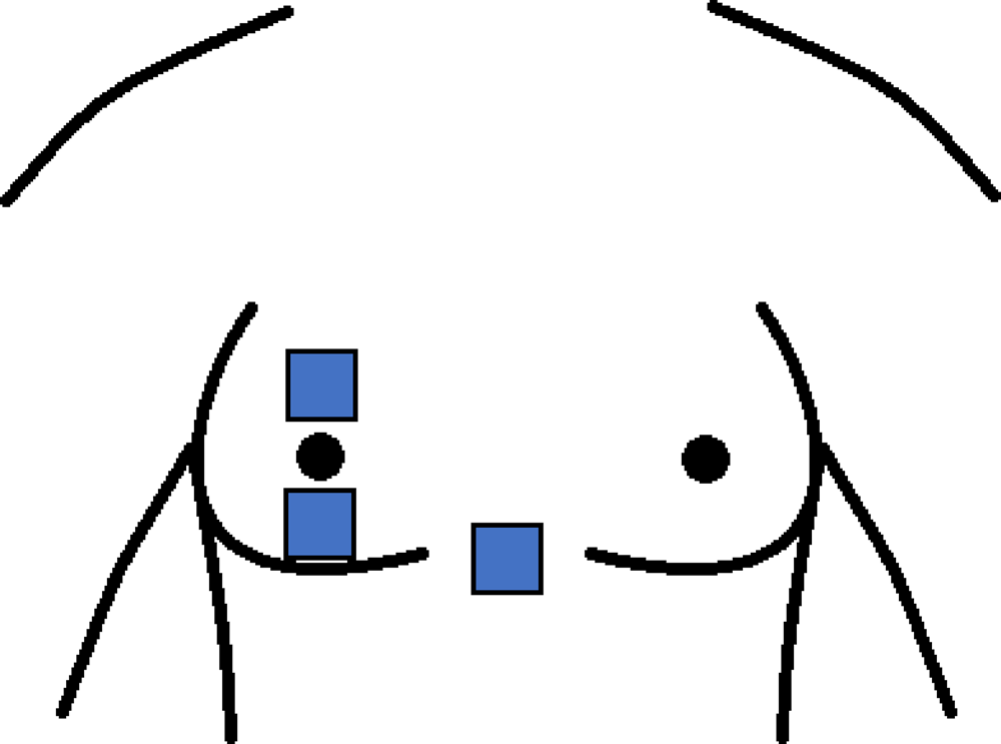
Sweat absorbent patch and iodine paper contact locations: above nipple, below nipple, bra triangle. 3 × 3 cm patches.

To establish breast size‐dependent differences in sweat gland density and output, we designed an experiment that aimed to achieve a steady‐state of sweating through a submaximal exercise protocol performed in the heat (Figure 1). At the start of each trial, breast geometry was captured using white‐light scanning techniques; participants then performed a 50‐min run in a climatic chamber set to 32±0.6°C and 53±1.7% relative humidity, during which thermophysiological (e.g., *T*_core_ and respiratory gases) and perceptual parameters (e.g., rate of perceived exertion) were recorded. During the final 5 min of the run, sweat gland density and LSR were measured at three locations across the breast (Figure 2). These data were then used to establish the associations amongst BrSA, sweat gland density and output, and LSR.

The running pace of the submaximal exercise protocol was kept constant throughout the trial and was self‐selected by each participant in the initial 5 min to elicit an average rate of perceived exertion (RPE) of 13, that is, ‘somewhat hard’, using the Borg Scale (Borg, 1982) over the course of the run. A fixed‐RPE model was selected to resemble a real‐life scenario whereby individuals run to a self‐regulated steady‐state pace based on perceived exertion. Pilot data also indicated that the resulting exercise intensity, combined with the warm environmental conditions, provided a sufficient thermal stimulus to elicit steady state sweating for the evaluation of sweat gland density and output (i.e., the former being our primary outcome for this study).

This experimental model resulted in varying individual levels of metabolic heat production and related evaporative requirements for heat balance (*E*_req_), which are well‐known factors that contribute to individual variability in LSR (Cramer & Jay, 2015). On this basis, the protocol incorporated an assessment of expired respiratory gases for the calculation of *E*_req_, which was subsequently used in the evaluation of its relative contribution to individual changes in LSR, alongside that of BrSA (see Section 2.5 for details). We acknowledge that a potential limitation of this approach is that a self‐selected exercise intensity could have resulted in, for example, decreasing level of *E*_req_ for participants with larger BrSA. As such, our data analysis incorporated an evaluation of the relationship between individuals *E*_req_ values and BrSA (see Section 2.5 for details).

### 2.4 Experimental procedures

Four hours prior to arrival at the laboratory, participants were instructed to ingest a telemetric pill (e‐Celsius Performance pill, BodyCAP, Caen, France) to measure T_core_. Pills were ingested 4 h prior to the experimental session to allow sufficient transit time to the intestine. Participants were instructed to wear a wristband for the following 72 h to identify that they had swallowed a magnetic resonance imaging‐incompatible device. During the experimental session, data from the pill were sampled every 15 s to the receiver (EQ‐eViewer Performance monitor, BodyCAP). Furthermore, participants were also instructed to drink 500 mL of water 2 h prior to testing to ensure hydration.

Upon arrival to the laboratory participants provided a urine sample to measure urine specific gravity (Digital refractometer, Kern, Balingen, Germany). If urine specific gravity was >1.025 g/mL participants were provided with 500 mL of water and tested again after 30 min before proceeding with the protocol (Casa et al., 2005). Anthropometric measures of height and BrSA were taken at rest in a thermoneutral laboratory (∼23°C and ∼50% relative humidity). Height was measured on a wall stadiometer and BrSA was estimated using a white‐light scanner (EinScan H, Shining 3D Tech. Co. Ltd, Hangzhou, China). Firstly, reflective markers were placed around the breast border based on a validated breast volume model (Göpper et al., 2020). Participants were then asked to adopt a 4‐point prone position such that the breasts could freely hang away from the torso, thus allowing a scan of the entire breast skin surface, from which surface area could be extracted using MeshLab (Visual Computing Lab, CNR‐ISTI, Pisa, Italy).

Upon entry to the climate chamber, participants were instructed to fully undress behind a privacy curtain and dry nude body mass was measured on a precision scale (Kern 150K2DL; accurate to 0.005 kg). Following the body weight measurement, participants were instructed not to drink throughout the trial until being weighed post‐run. Whole body sweat loss (WBSL) was calculated as the difference between pre‐ and post‐exercise body mass. Post‐exercise mass was measured as participants stepped off the treadmill following the initial 45 min of running.

Once weighed, participants were equipped with a heart rate (HR) monitor (1 Hz; Garmin 935, Garmin Ltd, Olathe, KS, USA) at the forearm, and provided with standardised running shorts and a sports bra and, whilst wearing their own personal trainers and socks. Next the participants were instructed to self‐select a running pace to elicit an average RPE of 13, or ‘somewhat hard’ using the Borg Scale over the course of the run, which was recorded every 5 min. This treadmill speed was kept constant throughout the trial. The treadmill gradient was maintained at 0% throughout the run.

At minute 45 of the run, or once volitional cessation occurred, participants briefly stepped off the treadmill and were asked to step behind the privacy curtain and fully undress to take another nude measurement. Following this, the set‐up of LSR and metabolic data collection for the final 5 min of the run occurred. LSR was measured using a modified absorbent technique developed by Smith and Havenith (2011). Before each trial, a set of absorbent material patches (maximum absorption = 4655 ± 220 g/m^2^) were cut to size (9 cm^2^), individually sealed in ziplock bags, marked and weighed to the nearest 0.1 mg using a precision scale (PCB 350‐3, Kern). Three sites across the chest were assessed including 3 cm above areola top, 3 cm below areola bottom, and the xyphoid process (‘bra triangle’) (Figure 2). Patches were affixed to a plastic insulating border then to the skin using a waterproof film dressing (Tegaderm, 3M, Saint Paul, MN, USA) to prevent the evaporation of sweat during the test periods. For each sample, the test site was wiped dry by the researcher, the patch was affixed to the skin and subsequently held in place with a dry bra. Following patch application, the participant donned the dry bra, and was equipped with the gas exchange mask prior to completing the final 5 min run. Following this, the participant stepped off the treadmill, the mask was removed and the absorbent patch was removed and sealed. To ensure a standardised patch application time for all participants, but also allow sufficient time for the mask set up and donning and doffing of clothing, the absorbent patch was affixed for 10 min for all participants. After exactly 10 min, the patches were removed, quickly sealed in ziplock bags, and re‐weighed. LSR (g/m^2^/h) was calculated using the difference in pre‐ to post‐patch weight, divided by the patch surface area and duration of application.

Oxygen consumption (${\dot{V}}_{O_{2}}$) and carbon dioxide (${\dot{V}}_{CO_{2}}$) production were measured by indirect calorimetry using a calibrated gas exchange analyser (Quark CPET, Cosmed, Rome, Italy) with a breathing mask. The mask was carefully placed over the mouth and nose to avoid air leakage and worn during the last 5 min of the running protocol. We opted for this sampling approach to eliminate the impact that wearing a mask throughout the whole trial could have had on running style and efficiency, the latter being a potential modulator of thermoregulatory responses (Smoljanić et al., 2014). In further support of the reliability of the measurements, we performed pilot studies with mask wearing throughout the 50‐min run to compare respiratory gas exchange levels between the initial 45 min and the final 5 min post mask wearing and confirmed that these levels were equivalent.

Partitional calorimetry was used to calculate evaporative requirement for heat balance ($E_{\mathrm{req}}$ in W/m^2^) over the last 5 min of running using the following formula (Parsons, 2007):

$$\;E_{\mathrm{req}}=H_{\mathrm{prod}}-H_{\mathrm{dry}}-H_{\mathrm{resp}}\left[W/m^{2}\right]$$

where $H_{\mathrm{prod}}$ is the rate metabolic heat production, $H_{\mathrm{dry}}$ is the rate of dry heat exchange and $H_{\mathrm{resp}}$ is respiratory heat exchange.

$H_{\mathrm{prod}}$ is calculated as the difference in metabolic rate (*M*) and external work rate (0 J as the treadmill has no incline):

$$M=\frac{\left(\left(\left((0.23\;\times \;\mathrm{RER})\;+\;0.77\right)\;\times \;5.88\right)\;\times \;\;\times \;60\right)}{\mathrm{BSA}}$$

where ${\dot{V}}_{O_{2}}$ is the oxygen consumption in L/min (STPD), RER is the respiratory exchange ratio (${\dot{V}}_{CO_{2}}$/${\dot{V}}_{O_{2}}$) and BSA is body surface area calculated using the following equation (Du Bois & Du Bois, 1916):

$$BSA=Wt{\left[kg\right]}^{0.425}\times Ht{\left[cm\right]}^{0.725}\times 0.007184.$$

$H_{\mathrm{dry}}$ is calculated as:

$$H_{\mathrm{dry}}=C+R\left[W/m^{2}\right]$$

$$C=h_{c}\left(T_{\mathrm{sk}}-T_{a}\right)\;\left[W/m^{2}\right]$$

$$R=h_{r}\left(T_{\mathrm{sk}}-T_{r}\right)\;\left[W/m^{2}\right]$$

where *C* and *R* represent convective and radiant heat exchange, respectively, *T*_a_ is ambient temperature (°C), *T*_sk_ is skin temperature (°C), measured using wireless thermistors (iButtons, Maxim, San Jose, CA, USA), *T*_r_ is the mean radiant temperature (°C), which was assumed to be equivalent to ambient temperature in the laboratory setting, *h*_c_ is the convective heat transfer coefficient, and *h*_r_ is the radiant heat transfer coefficient. Further details on these equations and assumptions are reported by Parsons (2007).

Following cessation of the run and immediately after removal of the absorbent pads, heat‐activated sweat gland density was measured non‐invasively using the modified iodine technique (Gagnon et al., 2012). Cotton paper patches were cut to 3 × 3 cm squares and impregnated with iodine. The skin was blotted dry to move excess water, then the iodine paper firmly pressed to the skin for ∼5 s. Sweat from active sweat glands appeared as small blue dots on the iodine infused paper. The paper was sealed in air‐tight bags and scanned immediately after testing at a high resolution (600 dots/inch) then analysed using ImageJ (https://imagej.net/ij/index.html). This process is explained in detail by Gagnon et al. (2012). The use of 3 × 3 cm patches permitted detection of regional differences while maintaining good inter‐day reliability (Peel et al., 2022). Sweat output per gland was subsequently calculated as the ratio between LSR and sweat gland density at each tested site (Buono & Connolly, 1992). It should be noted that this technique does not measure sweat output per gland directly, but rather it estimates mean glandular output in a local area from the LSR and density of active glands.

All data collection was performed by female researchers (although participants were always given the option of a male or female chaperone throughout testing).

### 2.5 Statistical analysis

Normality testing using the Shapiro–Wilk test was performed for all datasets, and homoscedasticity was assessed using Levene's test for regression analysis. Data for $E_{\mathrm{req}}$, WBSL, ∆*T*_core_, HR, running speed and running time are presented descriptively. Statistical analyses were carried out using SPSS Statistics (version 28.1; IBM Corp., Armonk, NY, USA). Data are reported as the means and SD, and significance was set at *P* < 0.05.

It is important to note that not all our participants were able to complete the full 50‐min trial duration, as some participants required an earlier termination of the run due to volitional fatigue. Thus, we divided the study cohort into ‘finishers’ (*n* = 16) and ‘non‐finishers’ (*n* = 6) for the purpose of data analysis. Specifically, all participants in the XL bra category (*n* = 5) and one participant in the large bra category were unable to complete the full 45‐min run trial. Previous evidence indicated that full recruitment of sweat glands occurs after an average exercise duration of 8 min at a similar exercise intensity as the one utilised in this study (Kondo et al., 2001). As all participants ran for a minimum of 20 min (mean ± SD; 44.6 ± 9.6 min), all data were included (*n* = 22) for the analysis of sweat gland density. In contrast, previous evidence indicated that LSR increases linearly with exercise duration (Kondo et al., 2001). Therefore, due to the exercise duration effect, only ‘finishers’ (*n* = 16) were included in the LSR and sweat output per gland analysis. Furthermore, we conducted correlation analyses between individual *E*_req_ values (in W/m^2^) and BrSA in all participants, as well as in the ‘finisher’ cohort, to identify any potential association between varying *E*_req_ levels resulting from self‐selected exercise intensities and BrSA.

Skin site‐dependent differences in sweat gland density amongst the above and below nipple sites and the bra triangle were analysed using a one‐way repeated measures ANOVA. The Greenhouse–Geisser correction was applied if the assumption of sphericity had been violated. In the event of statistically significant main effects, *post hoc* analyses were conducted using the Bonferroni test. In the absence of differences between above and below nipple sites (effectively the breast sites), sweat gland density data for the above and below nipple sites were averaged to determine a cumulative ‘breast site’. At this point, and to address our primary outcome, Pearson's correlation analyses between BrSA and sweat gland density was used, separately for the breast site and bra triangle.

Second, and to address the secondary outcome, the same site‐related analyses (i.e., via a one‐way repeated measures ANOVA), and Pearson's correlation analyses (i.e., with BrSA), were performed using LSR and sweat output per gland data for both the (cumulative) breast site and bra triangle.

Finally, and in accordance with the previously cited biophysical role of $E_{\mathrm{req}}$ in contributing to individual variability in LSR (see Cramer & Jay, 2015), exploratory multivariate linear regression analysis was used to assess BrSA and $E_{\mathrm{req}}$ in W/m^2^ (i.e., as the independent variables) and LSR at the breast site (i.e., as the dependent variable). This analysis was designed to quantify the relative contribution of both BrSA and $E_{\mathrm{req}}$ in W/m^2^ to individual variance in LSR, under conditions of self‐selected exercise intensity that would have elicited natural variations in $E_{\mathrm{req}}$ in W/m^2^ amongst participants. Assumptions of multi‐collinearity between these variables were assessed and satisfied.

## 3 RESULTS

### 3.1 Descriptive exercise and physiological data

Exercise and physiological variables are presented in Table 2 for both finisher and non‐finisher participants.

**TABLE 2:** Mean values, standard deviation (SD), minimum and maximum values of evaporative requirement for heat balance in (*E*_req_) (W/m^2^), whole body sweat loss (WBSL), core temperature change (∆*T*_core_), heart rate, run speed and run time measured during the submaximal run in the heat.

	Finishers (*n* = 16)									Non‐finishers (*n* = 6)				
	Small (*n* = 6)			Medium (*n* = 5)			Large (*n* = 5)			Large (*n* = 1)		X‐Large (*n* = 5)		
	Mean	SD	Min–Max	Mean	SD	Min–Max	Mean	SD	Min–Max	Mean	Min–Max	Mean	SD	Min–Max
*E*_req_ (W/m^2^)	356	36.1	289–380	337	15.9	316–358	318	43.5	275–386	307	307	364	44.2	318–416
WBSL (g)	605	196	298–605	793	169	670 –1070	693	273	490–1155	465	465	400	123	245–545
∆*T*_core_ (^o^C)	1.6	0.5	0.8–2.4	2.0	0.5	1.5–2.7	1.4	0.4	1.1–1.9	1.3	1.3	0.9	0.4	0.4 – 1.3
Heart rate (bpm)	151	5	142–155	165	9	154–176	160	11	143–171	160	160	151	24	122–152
Running speed (km/h)	7.5	1.2	6.4–9.7	7.2	0.8	6.4–8.0	6.8	0.8	6.1–8.0	7.2	7.2	7.6	1.2	6.1–8.9
Run time (min)	45	0	45	45	0	45	45	0	45	30	30	29	6.5	15–35

*Note*: Split by bra size and finishers (*n* = 16) and non‐finishers (*n* = 6).

### 3.2 Test site‐dependent differences in sweat gland density, output and LSR

When considering data from all tested women (*n* = 22), sweat gland density ranged between 12.1 and 71.2 and between 10.3 and 59.0 glands/cm^2^ at the above and below nipple sites, respectively, and between 56.8 and 136.9 glands/cm^2^ at the bra triangle. A significant effect of test site on sweat gland density was observed (*P* < 0.001) with sweat gland density at the bra triangle (92.8 ± 21.4 glands/cm^2^) being ∼3 times greater than above the nipple (35.8 ± 19.2 glands/cm^2^, *P* < 0.001) and below the nipple (31.9 ± 16.0 glands/cm^2^, *P* < 0.001). No differences were found between above and below nipple sites (*P* = 0.155).

When considering data from finisher women (*n* = 16), LSR ranged between 8.3 and 35.9 and between 6.4 and 44.4 g/m^2^/h at the above and below nipple sites, respectively, and between 74.0 and 225.5 g/m^2^/h at the bra triangle. A significant effect of test site on LSR was observed (*P* < 0.001) with LSR at the bra triangle (122.5 ± 43.4 g/m^2^/h) being ∼6 times greater than above the nipple (20.8 ± 7.6 g/m^2^/h, *P* < 0.001) and below the nipple (21.2 ± 12.0 g/m^2^/h, *P* < 0.001). No difference was found between above and below nipple sites (*P* = 0.917).

When considering data from finisher women (*n* = 16), output per gland ranged between 1.04 and 2.94 and between 0.64 and 5.79 mg/h at the above and below nipple sites, respectively, and between 2.6 and 7.8 mg/h at the bra triangle. A significant effect of test site on output per gland was observed (*P* < 0.001) with output at the bra triangle (4.31 ± 1.45 mg/h) being ∼2 times greater than above the nipple (1.80 ± 0.54 mg/h, *P* < 0.001) and below the nipple (2.24 ± 1.36 mg/h, *P* = 0.007). No difference was found between the above and below nipple sites (*P* = 0.619).

### 3.3 Correlation of BrSA with *E*_req_, sweat gland density, output and LSR

**FIGURE 3:**
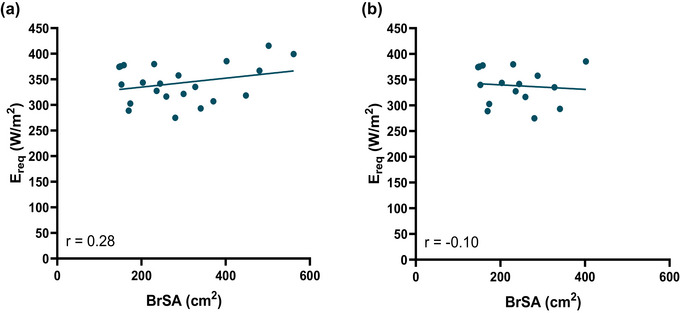
Relationship between breast surface area and evaporative requirement for heat balance (in W/m^2^) for (a) all participants (*n* = 22; used in sweat gland density analysis) and (b) the full 45‐min trial finishers (*n* = 16; used in LSR analysis).

First, we found no statistically significant correlation between BrSA and *E*_req_ when considering either all participants (*r* = 0.28, *P* = 0.201; Figure 3a) or the finisher participants (*r* = −0.10, *P* = 0.726; Figure 3b).

Second, given the lack of differences for all assessed variables between the above and below nipple sites, the data between these test sites was averaged, and from herein we report breast (average) sweat gland density, LSR and output data.

**FIGURE 4:**
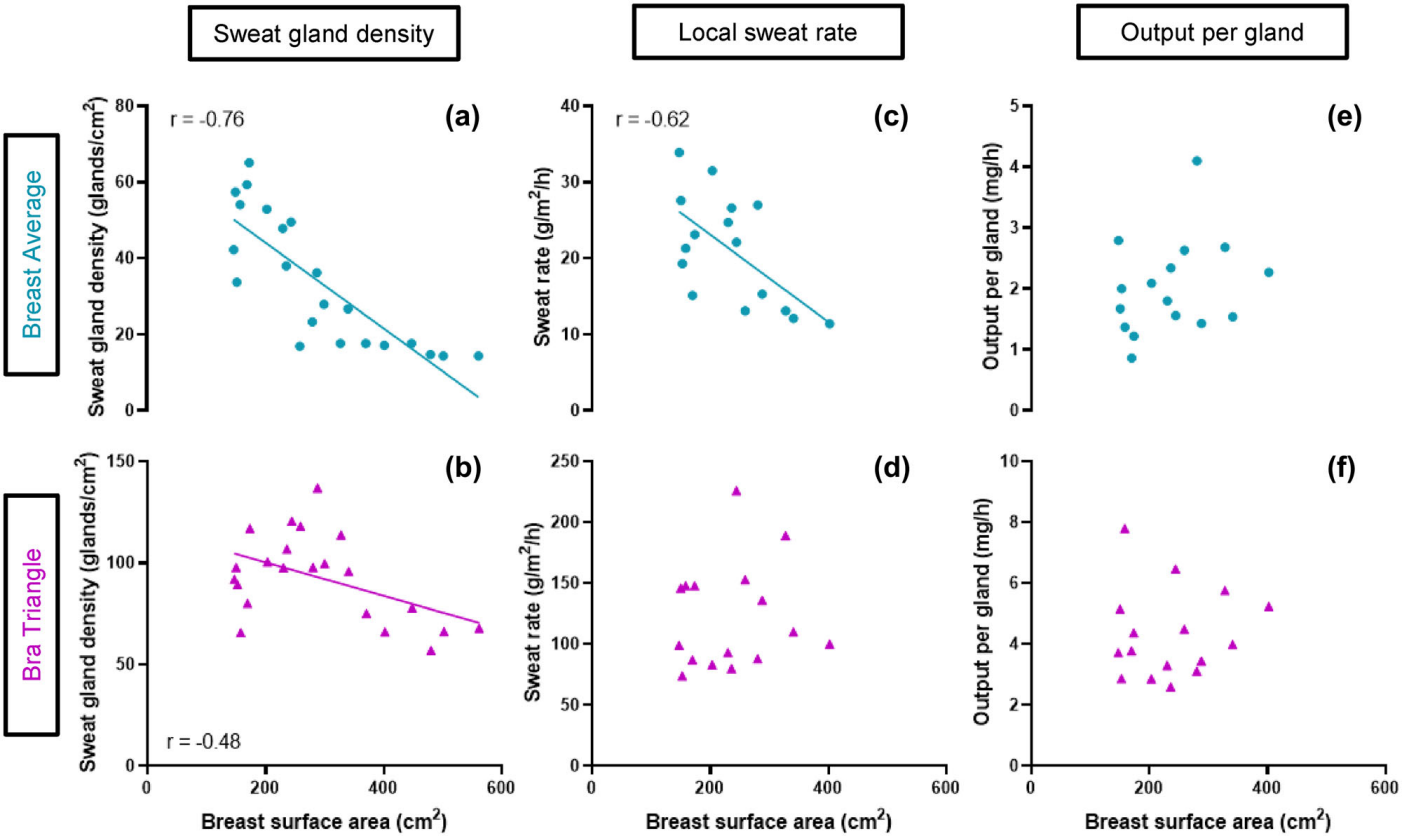
Relationship between sweat gland density (*n* = 22) (a, b), LSR (*n* = 16) (c, d) and output per gland (*n* = 16) (e, f) in two locations (breast average, bra triangle) relative to breast surface area.

When considering sweat gland density data (*n* = 22), a statistically significant negative correlation was found with BrSA across the breast (*r* = −0.76, *P* < 0.001; Figure 4a). A statistically significant, yet weaker correlation between BrSA and sweat gland density at the bra triangle was also observed (*r* = −0.48, *P* = 0.023; Figure 4b). Regarding LSR data (*n* = 16), a statistically significant negative correlation was found with BrSA across the breast site (*r* = −0.62, *P* = 0.011; Figure 4c). However, no significant relationship was found between BrSA and LSR at the bra triangle (*r* = 0.12, *P* = 0.654; Figure 4d). When considering sweat output per gland data (*n* = 16), no significant relationships were found between BrSA and output neither across the breast (*r* = 0.29, *P* = 0.279; Figure 4e) nor at the bra triangle (*r* = 0.05, *P* = 0.842; Figure 4f).

### 3.4 Multiple regression for LSR

The multiple regression model indicated that together BrSA and $E_{\mathrm{req}}$ (in W/m^2^) explained 40% of the total variance (*P *= 0.036) in LSR at the breast (Table 3). Out of the total variance explained, ∼33% was determined by BrSA (*P *= 0.015) and 7% by $E_{\mathrm{req}}$ in W/m^2^ (*P* = 0.538).

**TABLE 3:** Multiple regression model for changes in local sweat rate at the breast average site (*n* = 16).

	*b*	SE	*P*	Tolerance	$r^{2}$
Constant	24.955	15.959	0.142	1.564	
BrSA ($cm^{2}$)	−0.056	0.020	0.015*	−2.802	32.6%
$E_{\mathrm{req}}$ (W/$m^{2})$	0.028	0.044	0.538	0.632	7.4%

*P < 0.05. SE, standard error.

## 4 DISCUSSION

In relation to our primary aim, the results of this study confirmed our hypothesis that sweat gland density over the breast decreased linearly with increasing BrSA in healthy young to middle‐aged women. In relation to our secondary aim, our findings also confirmed our secondary hypothesis that sweat output per gland did not vary with BrSA, thereby leading to decreasing LSR with increasing BrSA. Of note, individual differences in BrSA explained a greater proportion of variance (i.e., ∼33%) in breast LSR than *E*_req_ in W/m^2^ (i.e., ∼7%), albeit this finding should be considered within the range of natural variation in *E*_req_ achieved in this study (i.e., min to max *E*_req_: 275–386 W/m^2^). In addition to our initial hypotheses, our results also indicated that, irrespective of BrSA, the breast had lower sweat gland density and output per gland than the bra triangle. Altogether, our findings confirm an established relationship between sweat gland density and LSR and extends this finding to the female breast, highlighting the relationship between breast morphology and sweat gland density and output, which has valuable implications for our understanding of sweat management requirements for sport bras.

Historically, regional sweat gland density has been investigated across large body regions (Kawahata, 1939; Kuno, 1956; Szabo, 1958). For example, early work by Kawahata (1939) investigated regional sweat gland density over the head (average sweat gland density: 260 glands/cm^2^), neck (222 glands/cm^2^), trunk (114 glands/cm^2^), upper extremities (114 glands/cm^2^) and lower extremities (100 glands/cm^2^). These investigations provided reference values, and they highlighted regional differences in sweat gland density across the human body. However, these studies had limited consideration of sex‐related differences and of the impact of body size/surface area across unique body parts such as the female breast. In this respect, the sweat gland densities that we observed for the female bra triangle (i.e., 92.8 ± 21.4 glands/cm^2^), that is, an area of the trunk presenting minimal breast development‐dependent variation in surface area, mostly corroborate with those previously measured in the trunk region by Kawahata (1939) (i.e., 114 glands/cm^2^). Yet, when considering the breast, we found that sweat gland densities decreased with increasing BrSA from a maximum of ∼71 glands/cm^2^ (i.e., in our smallest breasted women) to a minimum of ∼10 glands/cm^2^ (i.e., in our largest breasted women). This finding indicated that, while our smallest breasted women (i.e., BrSA = ∼168 cm^2^) presented sweat gland densities over the breast that were only slightly lower (i.e., maximum of 71 gland/cm^2^) than those measured at the bra triangle (and at the trunk, by Kawahata (1939)), sweat gland density in our largest breasted women (i.e., BrSA = ∼561 cm^2^) approached some of the lowest sweat gland density values ever reported across the body (Taylor & Machado‐Moreira, 2013). It has been previously reported that the maximum number of sweat glands that our skin contains is achieved by 2 years of age (Kuno, 1956). Hence, our findings provide compelling evidence that as the skin of the female breast stretches during the breast‐growth period occurring during puberty, sweat glands become less densely populated locally and in proportion to the extent of the breast growth.

It should be noted that our data also indicated a (weak) relationship between sweat gland density and BrSA at the bra triangle. However, this relationship may be primarily dependent on the observed strong and positive correlation between BrSA and BSA. Put in context, our larger breasted women also tended to be individuals with larger BSA. Hence, the size‐dependent mechanism associated with sweat gland density that we clearly observed at the breast, may have partly applied at the bra triangle of larger women (i.e., who exhibited reduced sweat gland density at the bra triangle likely due to greater skin stretch). This observation demonstrates a more general size‐dependent mechanism associated with BSA and sweat gland density reported in humans, irrespective of sex (Bar‐Or et al., 1968; Best et al., 2023).

In relation to the secondary aim of this study, we found that LSR at the breast also decreased linearly with increasing BrSA. This response was primarily driven by the fact that, while sweat gland density over the breast decreased with increasing BrSA, sweat output per gland remained predominantly constant. It therefore appears that in the case of the breast, sweat output per gland is not upregulated to accommodate a size‐dependent change in the density of sweat glands across the female breast. In the present study, the BrSA of participants within the small to extra‐large bra categories accounted for between 1% and 2.2% of participants’ whole‐body surface area. This means that the 7‐fold reduction in active sweat gland density that we observed between the smallest and largest breasts (see Fig. 3a) occurred across a very small portion of whole‐body surface area. Differences in local evaporative capacity between larger and smaller breasted women did not translate to differences in core temperature changes. Therefore, it can be inferred that the observed reduction in active sweat gland density in larger breasts was not accompanied by a greater drive for sudomotor output, hence a consequent (and observed) lack of upregulation of LSR at the breast. It should also be noted that sweat rates at the breast are generally low in comparison to other body parts, for example areas of the highest sweat rates on the female body (upper back, dorsal foot, bra triangle) produce 139–223 g/m^2^/h (Smith & Havenith, 2012) compared to 21 g/m^2^/h seen here at the breast. Hence, it appears that female breasts may, on the whole, play a relatively limited role in the regulation of whole‐body heat balance. Nevertheless, future studies should consider experimental designs where the independent role of sweat gland density and output over the breast on increases in core temperature can be assessed more directly.

As previously noted, we recognise the LSR findings presented in this study should be considered through the lens of an experimental protocol that was not designed with the primary purpose of isolating the independent effect of BrSA on LSR (i.e., in that case the administration of an exercise intensity at a fixed *E*_req_ expressed in W/m^2^ would have been more appropriate). Nevertheless, a relevant finding of this study is that individual differences in BrSA explained a greater proportion of variance (i.e., ∼33%) in breast LSR than *E*_req_ in W/m^2^ (i.e., ∼7%). Drivers of variability in LSR have previously been investigated and modelled by Cramer and Jay (2015), who concluded that *E*_req_ (in W/m^2^) plays a prominent role in describing individual variations in LSR. This is in contrast with our findings over the breast, and it may highlight site‐dependent differences in the biophysical drivers of LSR (note: most data for Cramer and Jay's model arise from measurements at the forearm). However, it is important to note that in our study, the range of *E*_req_ in W/m^2^ achieved by our finisher participants (i.e., *n* = 16, *E*_req_ = 275–386 W/m^2^) was the result of the natural variation associated with participants having to self‐select a running speed at a fixed RPE. Our *E*_req_ range was half as large as that employed in the study by Cramer and Jay (2015) (i.e., *E*_req_ in the range of 137–350 W/m^2^) and it also reached beyond their absolute maximum. These considerations may partly explain our observation of a less prominent role of *E*_req_ in driving LSR at the breast. In support of this, we note that the linear relationship between *E*_req_ and LSR in the study by Cramer and Jay (2015) becomes much more variable at higher levels of *E*_req_ (i.e., >200 W/m^2^). Hence, it cannot be excluded that a more complex interplay amongst biophysical, physiological and morphological factors in driving variability in LSR at the breast may occur when exercising at higher *E*_req_ (in W/m^2^). It is of course important to note that 60% of the variance in LSR at the breast remains unexplained in this study, and it is reasonable to hypothesise that variability in age (Larose et al., 2013), heat acclimation level (Lorenzo & Minson, 2010) or training status (Armstrong & Maresh, 1998; Armstrong & Armstrong, 2000) amongst our cohort may contribute to such unexplained variance. Furthermore, and as opposed to the sweat gland density analysis, only ‘finisher’ participants were included in the LSR analysis (which constituted a secondary aim). This may somewhat limit the generalisability of our LSR findings to very large‐breasted women. Nevertheless, we believe that the role of body morphology and associated sweat gland density and output as important contributors to variations in LSR should be emphasised, particularly across regions such as the female breast.

It is worth nothing that, as far as the authors are aware, no BSA prediction equation currently exists which accounts for BrSA in women. For example, equations to better estimate BSA in specific populations, including women, have been recently published (Ashby‐Thompson et al., 2020), and they (somewhat) overcome previous limitations with, for example, Du Bois and Du Bois equations, particularly in larger individuals. Yet, these corrected equations still do not account for variation in BrSA in women. The directly measured range of BrSA in our study corresponded to ∼1% to 2.2% of total BSA (as calculated by Du Bois and Du Bois equations). Hence, it may be reasonable to suggest that the range of estimation error in routine BSA calculations (i.e., using height and weight parameters only) could be up to ∼2% in women. This observation may be relevant when prescribing exercise intensities at a fixed *E*_req_ in W/m^2^ for the comparison of LSR responses in different groups that include women of varying breast sizes. Indeed, it would be reasonable to suggest that the BSA estimation error may translate into *E*_req_ levels that could differ by up to 2% (e.g., ±4 W for a 1.8 m^2^ individual exercising at 100 W/m^2^). While such a difference in *E*_req_ is unlikely to translate in meaningful differences in LSR, the potential for this estimation error is worth considering in the context of the unexplained variance in LSR after accounting for *E*_req_ in W/m^2^ (i.e., unexplained variance = ∼40%; see Cramer and Jay (2015)).

Finally, we note that irrespective of BrSA, the breast had lower LSR than the bra triangle due to presenting both a lower sweat gland density and lower sweat output per gland. These regional differences in LSR are in line with those previously reported by Smith and Havenith (2012), who also observed lower LSR at the breast than bra triangle (i.e., bra triangle LSR = 139 g/m^2^/h vs. upper breast LSR = 11 g/m^2^/h). Our findings build upon this previous work as we have now demonstrated that these regional differences are driven by the presence of both a lower sweat gland density and lower sweat output per gland over the breast. This observation highlights further the complex relationship amongst body morphology, body regional differences, and sweat gland density and output across the female body.

Beside their fundamental relevance to understanding female‐specific body temperature regulation, our findings carry important applied implications to sport bra design and sex‐specific clothing evaluations with thermal manikins. Models of human thermophysiology have been evolving from simple two‐factor models (core and skin compartments; Gagge, 1971) to multi‐segmented models to reflect whole body shape from a heat transfer and sweat production approach (Fiala et al., 2012; Smith & Havenith, 2012). Thus, our findings could inform the development of female‐specific models that incorporate a more evidence‐based approach to the investigation of heat transfer and sweat production across the female breast. Better understanding of how breast size impacts local sweat gland density and output could support the design of sports bras that serve a wider range of consumers with tailored solutions based on individual needs regarding heat dissipation and skin wetness‐dependent comfort (Filingeri et al., 2015).

### 4.1 Conclusion

Individual differences in BrSA were observed to modulate both sweat gland density and LSR in healthy young to middle‐aged females. Our findings confirm the established relationship between sweat gland density and body morphology, and they further extend this observation to the female breast, highlighting a similar relationship between BrSA and sweat gland density and LSR. This observation provides novel insights on individual differences in the anatomy and physiology of sweating at the female breast, which may impact the thermoregulatory responses (both autonomic and behavioural) of women of different breast sizes. Furthermore, our results have important applied implications to inform the design of sports bras that meet the thermal needs of women with a range of breast sizes. This may ultimately increase clothing comfort and performance, thus reducing barriers to maintaining an active lifestyle in women of all bra sizes.

## AUTHOR CONTRIBUTIONS

Hannah Blount, Davide Filingeri and Peter Worsley conceived the initial outline for the article. Acquisition was completed by Hannah Blount, Alessandro Valenza and Jade Ward. Hannah Blount, Silvia Caggiari, Davide Filingeri, and Peter Worsley contributed to interpretation. Hannah Blount, Davide Filingeri, Peter Worsley contributed to drafting the manuscript. All authors approved the final version of the manuscript and agree to be accountable for all aspects of the work in ensuring that questions related to the accuracy or integrity of any part of the work are appropriately investigated and resolved. All persons designated as authors qualify for authorship and only those who qualify for authorship are listed.

## CONFLICT OF INTEREST

The authors declare they have no conflicts of interest.

## Data Availability

Data will be made available upon publication at the University of Southampton data repository (PURE; URL to be activated upon publication).
